# Application of receiver operating characteristic curve in the assessment of the value of body mass index, waist circumference and percentage of body fat in the Diagnosis of Polycystic Ovary Syndrome in childbearing women

**DOI:** 10.1186/s13048-016-0260-9

**Published:** 2016-08-24

**Authors:** Pan Dou, Huiyan Ju, Jing Shang, Xueying Li, Qing Xue, Yang Xu, Xiaohui Guo

**Affiliations:** 1 Department of Clinical Nutrition, Peking University First Hospital, Beijing, China; 2 Center of Reproduction and Genetics, Peking University First Hospital, Beijing, China; 3 Department of Biostatistics, Peking University First Hospital, Beijing, China; 4 Department of Endocrinology, Peking University First Hospital, Beijing, China; 5 No.7, Xishiku Road, Xicheng District, Beijing, 100034 People’s Republic of China

**Keywords:** Childbearing age, Polycystic ovary syndrome, Obesity, Body mass index, Waist circumference, percentage of body fat, Receiver operating characteristic, Area under the curve

## Abstract

**Background:**

There are various parameters to analyze obesity, however, no standard reference to predict, screen or diagnose PCOS with various obesity parameters has been established, and the accuracy of these parameters still needs to be studied.This study was to use the receiver operating characteristic (ROC) curve to explore the different values of three obesity parameters, body mass index (BMI), waist circumference (WC) and percentage of body fat (PBF) in the diagnosis of polycystic ovary syndrome (PCOS) in Chinese childbearing women.

**Methods:**

Three hundred patients who were diagnosed with PCOS at Center of Reproductive Medicine and Genetics of Peking University First Hospital were enrolled in this study, and 110 healthy age-matched women were enrolled as controls. The characteristics of BMI, WC and PBF in PCOS patients were analyzed.

**Results:**

Compared with the control group, all the three obesity parameters were significantly increased in PCOS group. In terms of ROC area under the curve, WC > PBF > BMI, and they were all significantly different from those of the control. At a cut-off point of 80.5 cm, WC has a sensitivity of 73.6 % and a specificity of 85 % in diagnosis of PCOS; At a cut-off point of 29 %, PBF has a sensitivity of 88.2 % and a specificity of 57.7 % in diagnosis of PCOS; and at a cut-off point of 26.6 kg/m^2^, BMI has a sensitivity of 54.5 % and a specificity of 98 % in diagnosis of PCOS.

**Conclusion:**

WC, BMI and PBF are valuable in screening and diagnosis of PCOS in Chinese childbearing women. PBF can be used to screen PCOS as it has a better sensitivity, while BMI can be used in the diagnosis of PCOS as it has a better specificity.

## Background

Polycystic ovary syndrome (PCOS) was first reported by Stein and Leventhal [1] in 1935, so it is also called Stein-Leventhal syndrome. It is the most common female endocrine and metabolic disorder, and its incidence rate is about 5.6 % among women aged 19–45 years old in China [2]. PCOS is a complex disease with high clinical heterogeneity, excess androgen production and elevated serum luteinizing hormone (LH) are its serological features. It is a major cause of infertility in women. Studies from different countries have shown that the comorbidity rate of obesity in patients with PCOS was 30–70 % [3, 4]. The reproductive, endocrinological and metabolic disorders (except hirsutism) in obese PCOS patients are more severe than non-obese patients, and the long-term risk of the incidence of metabolic syndrome, type 2 diabetes mellitus (DM), cardiovascular disease (CVD) as well as breast cancer, endometrial cancer and other complications was also increased exponentially [5]. There are various parameters to analyze obesity, however, no standard reference to predict, screen or diagnose PCOS with various obesity parameters has been established, and the accuracy of these parameters still needs to be studied. This study was designed based on the Rotterdam PCOS consensus criteria, to explore the accuracy and best cut-off points of three obesity parameter, i.e., body mass index (BMI), waist circumference (WC) and percentage of body fat (PBF), in PCOS diagnosis, as well as to compare their sensitivity and specificity to provide basis for rational application of obesity parameters in prediction, screen and diagnosis of PCOS among high-risk populations.

## Methods

### Study subjects

Three hundred study subjects who were diagnosed with PCOS at Reproduction and Genetic Center of Peking University First Hospital from June 2015 to January 2016 were selected. Patients were did not take any hormone medication within the past 3 months. 110 healthy women of childbearing age who have normal menstruation period and biphasic basal body temperature were selected as normal control. The ages of the two groups were matched to each other. This study was approved by the Ethics Committee of China Registered Clinical Trial and all the study subject have signed informed consent to voluntarily participate the study.

### PCOS diagnostic criteria

Based on the American Society for Reproductive Medicine (ASRM) Rotterdam Revised Diagnostic Criteria, a patient can be with diagnosed as PCOS if two of the following three criteria are met: (1) no ovulation or irregular ovulation; (2) clinical (such as hirsutism, acne) or biochemical evidence associated with elevated androgen levels; (3) enlarged ovaries, each side has 12 or more small follicles with a diameter of at least 2 ~ 9 mm; plus exclusion of hyperlactatemia and other metabolic diseases that produce high-level of androgen such as Cushing’s syndrome, congenital adrenal hyperplasia, ovarian or adrenal tumors.

### Anthropometric measurements

Measurements were carried out in the hospital’s outpatient clinic, performed by the same observer in accordance with the provisions of WHO. For measurement of height (m), the examinee was required to be barefoot, the rear point of the feet, hip and the rear point of head were on the same vertical line, the measurement value was approximated to the nearest 0.5 cm; for measurement of body weight (kg), electronic scale was used, and the examinee was required to be fasted overnight and urine and stool were emptied, only underwear was allowed, the measurement values was approximated to the nearest 100 g, BMI was calculated with the formula: weight (kg)/(height)^2^ (m^2^); for measurement of waist circumference, the examinee was required to stand upright with two feet apart 25 ~ 30 cm, so that the weight is evenly distributed on both legs, the waist circumference was measured in a horizontal level through the midpoint that links the iliac crest and the lower margin of the 12th rib (the measurement tape was placed close to the skin, but cannot repress the soft tissue), the measurement values was approximated to the nearest 1 mm.

### Body composition measurement

The body composition of the two groups was measured using bioelectrical impedance body composition analyzer (Multi-frequency bioelectrical impedance analyzer NQA-PI). On the test day, the examinee was asked to minimize eating and drinking, no tense activity was allowed within 6 h before the test to avoid its affection on body composition measurements. During measurement, the examinee was asked to take off socks, standing on the test bench with the body relaxed, with both feet on the foot electrodes and both hands holding firmly on the hand electrodes, then basal metabolic rate, total water, the amount of non-fat tissue, muscle mass, body fat mass, and percentage of body fat (PBF) were measured.

### Statistical analysis

SAS 9.3 software was used in statistical analysis. 1). Measurement data were shown as $\overline{x}\pm s,$ independent sample t test or Wilcoxon test was used to compare difference between the two groups, P <0.05 was considered statistically significant; 2). Receiver operating characteristic curve (ROC curve) was drawn using Mann-Whitney method, and area under the ROC curve (area under curve, AUC) and Somers’ D were used to determine the overall accuracy of each predictor (area under the curve ≥0.5 was considered to have diagnostic value, the larger the area, the larger the value); the optimal cut-off points for BMI, WC, PBF to predict PCOS was determined according to Youden index maximum points; 3). The cut-off point where the sensitivity reaches 90 % was determined as the reference standard for screening PCOS with BMI, WC and PBF; 4). The cut-off point where the specificity reaches 90 % was determined as the reference standard for diagnosis of PCOS with BMI, WC and PBF.

## Results

### Comparison of Age, BMI, WC and PBF between the two groups

**Fig. 1:**
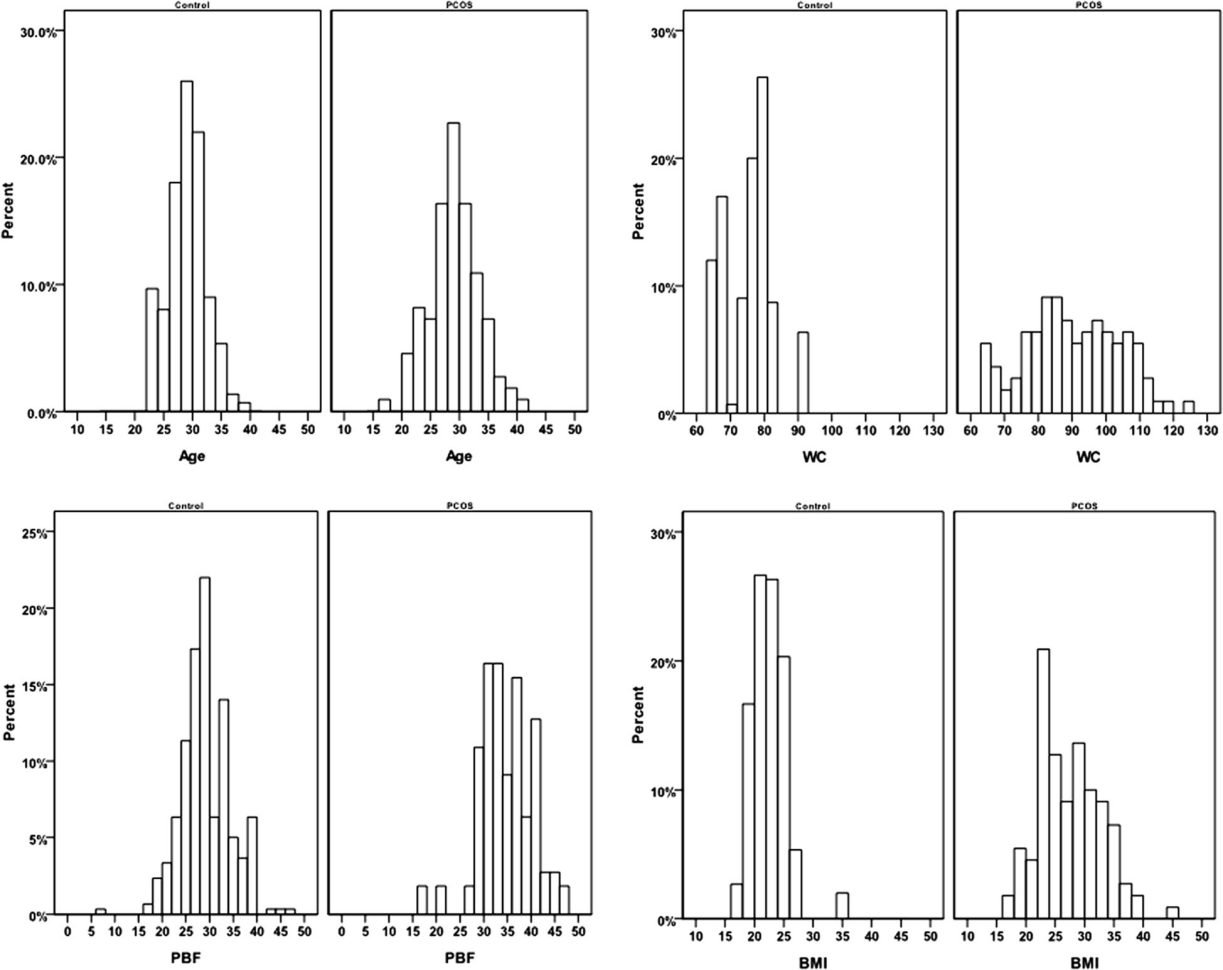
The percentage histogram of age, WC, PBF and BMI between PCOS and normal control groups

Comparison was performed using independent samples *t* test or Wilcoxon test. The ages of the PCOS group were between 16 to 40 years with mean 28.55 ± 4.27 years; the ages of control group were between 23 to 38 years with mean 28.53 ± 3.26 years. There was no significant difference between the two groups (Table 1). As shown in Table 1 and Fig. 1, compared with the healthy control group, all three parameters, WC (89.67 ± 13.93 vs 75 ± 7.11), PBF (34.69 ± 5.72 vs 29.12 ± 5.05) and BMI (27.51 ± 5.37 vs 22.55 ± 2.9) in PCOS group were significantly increased (*P* =0.000 for all comparisons), indicating that these three parameters are valuable in facilitating the screening or diagnosis of PCOS.

**Table 1:** Comparison of age, WC, PBF and BMI between control and PCOS groups

variables	Statistical parameters	Control group (*n* = 300)	PCOS group (*n* = 110)	*P*
Age (y/o)	$\overline{x}\pm s$	28.53 ± 3.26	28.55 ± 4.27	0.908
	Median	28.5 (27,30)	28 (26,31)	
	Range (Max, Min)	15 (23,38)	24 (16,40)	
WC (cm)	$\overline{x}\pm s$	75 ± 7.11	89.67 ± 13.93	0.000
	Median	75 (67,79)	89 (80,100)	
	Range (Max, Min)	28 (64,92)	60 (64,124)	
PBF (%)	$\overline{x}\pm s$	29.12 ± 5.05	34.69 ± 5.72	0.000
	Median	28.58 (26,32)	34.19 (30.98,38.27)	
	Range (Max, Min)	40.1 (6.55,46.65)	31.32 (16.11,47.43)	
BMI(kg/m^2^)	$\overline{x}\pm s$	22.55 ± 2.9	27.51 ± 5.37	0.000
	Median	22.81 (20.39,24.01)	27.26 (23.36,31.61)	
	Range (Max, Min)	16.92 (17.46,34.37)	26.77 (17.46,44.23)	

*Max* Maximum, *Min* Minimum; *y*/*o* years old

### ROC curves of BMI, WC and PBF for diagnosing PCOS

**Fig. 2:**
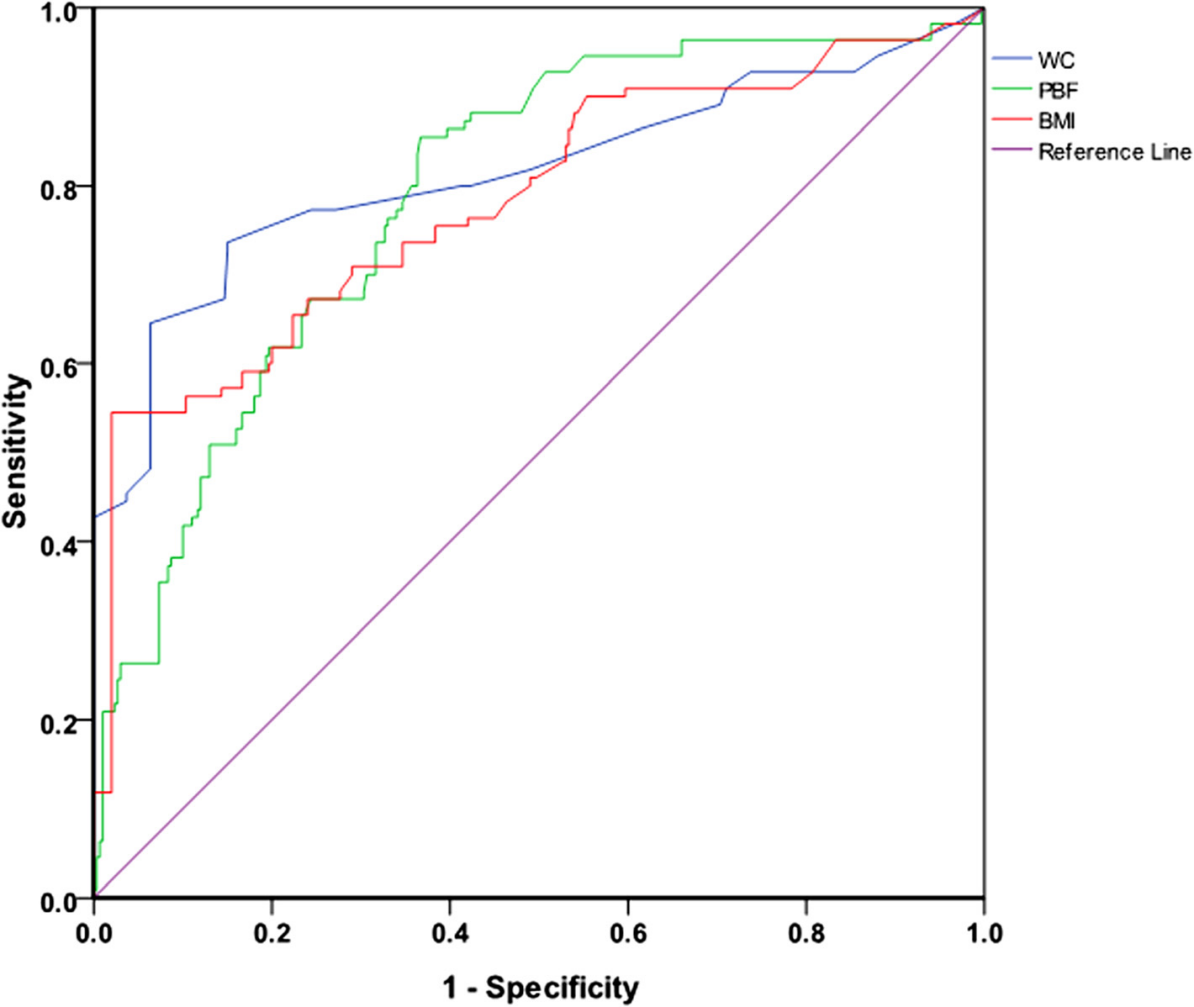
ROC curve of WC, PBF and BMI for the diagnosis of PCOS

The Rotterdam criteria was used as the gold standard when to evaluate the accuracy of BMI, WC and PBF to predict PCOS. Figure 2 is the ROC curve of the three obesity parameters for diagnosing PCOS, Table 2 is the area under the ROC curve of the three parameters for diagnosing PCOS. The results in Table 2 show that in general, the mean AUC for WC to diagnose PCOS is 0.814 with a standard error of 0.029 (*P* < 0.001 compared with 0.5 which was set as a standard comparison for AUC, please refer to the Statistical Analysis section); the mean AUC for PBF to diagnose PCOS is 0.789 with a standard error of 0.025 (*P* < 0.001 compared with 0.5 ); the mean AUC for BMI to diagnose PCOS is 0.782 with a standard error of 0.028 (*P* < 0.001 compared with 0.5). Each of the two groups was further divided into three subgroups according to age: <26 y/o group, 26–31 y/o group and >31 y/o group, and the data were further analyzed according to age groups. It was shown that the AUCs of all the subgroups of the three obesity parameters were all significantly larger than 0.5 (*P* <0.001). Comparisons were also performed among the three parameters and there is no significant difference between any of them, meaning that there is no better predictor for PCOS among them.

**Table 2:** AUC of ROC of WC, PBF and BMI to diagnose PCOS

Age (y/o)	Obesity parameter	AUC ± SE	95 % CI of AUC		Somers’ D	*P* ^a^	*P*
			Lower limit	Upper limit			
<26	WC (cm)	0.798 ± 0.068	0.665	0.930	0.596	<0.001	0.558^b^
	PBF (%)	0.769 ± 0.060	0.650	0.887	0.537	<0.001	0.526^c^
	BMI (kg/m^2^)	0.817 ± 0.059	0.701	0.933	0.634	<0.001	0.134^d^
26–31	WC (cm)	0.787 ± 0.0423	0.704	0.8700	0.574	<0.001	0.551^b^
	PBF (%)	0.765 ± 0.0361	0.694	0.8355	0.529	<0.001	0.544^c^
	BMI (kg/m^2^)	0.768 ± 0.0403	0.689	0.8472	0.537	<0.001	0.898^d^
>31	WC (cm)	0.889 ± 0.043	0.806	0.973	0.779	<0.001	0.001^b^
	PBF (%)	0.838 ± 0.042	0.756	0.920	0.676	<0.001	0.090^c^
	BMI (kg/m^2^)	0.773 ± 0.061	0.654	0.891	0.545	<0.001	0.138^d^
合计	WC (cm)	0.814 ± 0.029	0.758	0.870	0.629	<0.001	0.100^b^
	PBF (%)	0.789 ± 0.025	0.7397	0.838	0.577	<0.001	0.261^c^
	BMI (kg/m^2^)	0.782 ± 0.028	0.7265	0.838	0.564	<0.001	0.724^d^

^a^AUC compared with 0.5

^b^compared with BMI

^c^compared with WC

^d^compared with PBF

### The cut-off points and other features of the three obesity parameters to diagnose PCOS

ROC curves as mentioned above was used to select the best cut-off points of each of the three parameters to diagnose PCOS. As mentioned in the Statistical Analysis section, the best cut-off points for each of the three parameters to diagnose PCOS was determined based on the maximum point of Youden index. In general, the best cut-off point for WC to diagnose PCOS is 80.5 cm, and at that point, its sensitivity to diagnose PCOS is 73.6 %, and specificity is 85.0 %; the best cut-off point for PBF to diagnose PCOS is 29 %, and at that point, its sensitivity to diagnose PCOS is 88.2 %, and specificity is 57.7 %; the best cut-off point for BMI to diagnose PCOS is 26.6 kg/m^2^, and at that point, its sensitivity to diagnose PCOS is 54.5 %, specificity is 98.0 %. The results are shown in Table 3. In the age subgroups, the trend is similar: it seems that PBF has a better sensitivity and can be used to screen PCOS, while BMI can be used in the diagnosis of PCOS as it has a better specificity. The only exception is that in age subgroup of older than 31 y/o, WC has a better sensitivity than PBF (81.3 % vs 71.9 %).

**Table 3:** Cut-off points and other parameters of WC, PBF and BMI in diagnosis of PCOS

Age (y/o)	Obesity parameter	Cut-off point	Sensitivity (%)	Specificity (%)	Youden index	NPV (%)	PPV (%)
<26	WC (cm)	93.5	56.5	100	0.565	69.7	100
	PBF (%)	28.3	91.3	64.2	0.555	88.1	71.8
	BMI (kg/m^2^)	26.6	56.5	100	0.565	69.7	100
26–31	WC (cm)	80.5	69.1	84.5	0.536	73.2	81.7
	PBF (%)	29.9	85.5	60.9	0.464	80.8	68.6
	BMI (kg/m^2^)	26.5	45.5	98.9	0.444	64.5	97.6
>31	WC (cm)	81.0	81.3	93.2	0.745	83.3	92.3
	PBF (%)	33.0	71.9	83.6	0.555	74.8	81.4
	BMI (kg/m^2^)	26.6	68.8	94.5	0.633	75.2	92.6
Total	WC (cm)	80.5	73.6	85.0	0.586	76.3	83.1
	PBF (%)	29.0	88.2	57.7	0.459	83.0	67.6
	BMI (kg/m^2^)	26.6	54.5	98.0	0.525	68.3	96.5

## Discussion

Studies from different countries have shown that the incidence rate of obesity in patients with PCOS was 30–70 % [3, 4]. Obese PCOS patients tend to have more severe endocrinological, reproductive and metabolic disorders (except hirsutism), manifested by increases of total testosterone level, fasting glucose level, fasting insulin level, insulin resistance and lipid lvel, and decrease of sex hormone-binding globulin (SHBG) level [6]. The current view of the main mechanism of PCOS is the accumulation of excessive visceral fat tissue which causes insulin resistance through secretion of factors such as leptin [7], adiponectin, interleukin-6. This was followed by promotion of androgen synthesis by theca cells and inhibition of hepatic synthesis of SHBG, which together lead to increase of free testosterone concentration in the blood , and subsequently exacerbation of hyperandrogenism further [8]. In addition to affecting women’s reproductive function and pregnancy outcomes, the risk of metabolic syndrome, type 2 DM, CVD and breast cancer, endometrial cancer and other long-term complications in obese PCOS patients are also exponentially increased [5]. The main causes of obesity in PCOS patients are excessive daily intake of carbohydrate-rich, high-glycemic and high saturated fat diet, and too little exercise [9]. Mild weight loss in overweight or obese PCOS patients (a decrease of 5–10 %) could lead to decline in serum testosterone levels, also lead to return of normal ovulation cycle, improve pregnancy success rate [10–13] , and improve hormone, glucose and lipid metabolism disorders [14] and decrease the risk for CVD [15]. Therefore, physicians should not only intervene against PCOS’ clinical manifestations, but also should encourage weight loss in these PCOS patients to decrease long-term metabolic risk.

There are many parameters in analyzing obesity, among which, BMI, WC and PBF are the most widely used ones, but with different focus: BMI focus on evaluation of human density, WC focus on evaluation of human girth, PBF focus on evaluation of body fat [16]. The accuracy of using these three parameters to predict and diagnose obese PCOS still need to be assessed. In the meantime, due to the difference in race among different countries around the world, there is difference in detection rate of obesity when different cut-off points of obesity parameters were used, therefore, it is necessary to study the cut-off points of BMI, WC and PBF unique to Chinese women of child-bearing age with PCOS. This study was based on the Rotterdam diagnosis criteria for PCOS [17], where we analyzed the BMI, WC and PBF of 300 women ofchild-bearing age with PCOS who visited the Reproduction and Genetic Center in Peking University First Hospital from June 2015 to January 2016, as well as 110 cases of age-matched healthy women as normal control. We compared the sensitivity and specificity of the three obesity parameters in diagnosing PCOS, trying to provide evidence for reasonable use of obesity parameters in facilitating the diagnosis of PCOS among high risk population.

The results of this study showed that compared with the healthy control group, all three parameters in PCOS group, BMI, WC and PBF were significantly increased, consistent with previous reports [18], indicating the severity of overweight and obesity in patients with PCOS which leads to exacerbation of endocrine and reproductive metabolic disorders [19].

Our results also showed that, the AUC features of the three obesity parameters were greater than 0.5. AUC reflects the sensitivity and specificity of a certain index in general in the diagnosis of diseases, and the overall diagnostic accuracy of this indicator: 0.5 < AUC ≤ 0.7 means a low diagnostic accuracy, 0.7 < AUC ≤ 0.9 means medium diagnostic accuracy, 0.9 < AUC <1.0 means a high diagnostic accuracy [20]. This study demonstrated that these three parameters are all valuable for predicting obesity PCOS, and there was no significant difference among them. Previous studies show that, WC is superior in the accuracy of BMI and other parameters in predicting metabolic syndrome in China [21, 22], metabolic syndrome in PCOS patients in South Korea [23] and Brazil [24]. The reason, mainly is that obesity and overweight are two different concepts. BMI represents body mass and represents a proportional relationship between weight and height, and it cannot reflect the body fat content [25–28], and therefore it is not appropriate to use BMI alone to assess the degree of obesity. WC is an indicator of central obesity and central obesity is not correlated with human mass index, it mainly refers to the accumulation of visceral fat, thus it is a better predictor of risk of metabolic syndrome and cardiovascular disease [29], and WC is the simplest method of human body measurement. Results of this study suggest that at the time of the census of PCOS among childbearing women, it is recommended that the first choice is WC measurement since it is simple and has equal value in diagnosing PCOS.

Based on bioelectrical impedance principle, body composition analyzer can measure content of water in human body, as well as calculate PBF. It is an accurate, noninvasive and simple method to measure of the content and the proportion of human body fat [30]. Previous studies have shown that BMI showed high specificity and low sensitivity in the diagnosis of obesity, pointing out that BMI missed almost half of the obese population diagnosed by PBF [31–33]. Recent studies have shown that even with normal-weight, high PBF was also associated with insulin resistance [34], PBF is a better indicator to predict obesity [35], so more and more scholars began to study the value of measurement of body composition in obesity diagnosis [36–40]. The results of this study showed the AUC of PBF is not significantly different from the other two parameters, not consistent with previous findings [41]. Nevertheless, this study suggests that in the future, on the basis of PCOS sample, we can further dig into the value of body fat percentage measured using bioelectrical impedance method in the diagnosis of PCOS.

World Health Organization (WHO) [42] and the International Diabetes Federation (IDF) [43] have determined the cut-off point of BMI and WC in diagnosing obesity according to differences in the ethnic, race and morphology: For Chinese adult, 24 kg/m^2^ ≤ BMI <28 kg/m^2^ is defined as overweight, the BMI ≥ 28 kg/m^2^ is defined as obese; or female waist ≥80 cm is defined as overweight or central obesity; The normal range value of female PBF is 23 ± 5 % and PBF ≥ 30 % is defined as obese. Korean studies [23] showed that WC 80 cm was the best cut-off point for the best prediction of PCOS in Korean women; Brazilian studies [24] show WC 95 cm was the best cut-off point for the best prediction of PCOS in Brazilian women. The results of this study confirmed that, BMI 26.6 kg/m^2^, WC 80.5 cm and PBF 29 %, were the best cut-off point for diagnosing PCOS. The results of this study is similar to the studies with South Korea, which may be related to ethnic characteristics.

The results of this study also showed that, the three obesity parameters have different sensitivity and specificity. For example, whether in general or in age subgroups, BMI always has the best specificity among the three. While with only one exception in one age subgroup, PBF always has the best sensitivity among the three. These results indicate that, to screen for high-risk PCOS patients in Chinese women of reproductive age, PBF is the first choice as it is more sensitive, except in patients over 31 y/o, WC can be used as an alternative. So the cut-off point of PBF at 29 % can be used as reference standard for epidemiological investigation and preliminary screening of PCOS in the community, then the gold standard of Rotterdam criteria was used for confirmatory diagnosis. In this case, not only the risk factor of PCOS can be prevented earlier, but also avoid causing excessive pressure to the public, it can also adapt to the manpower burden in disease prevention and control, is a reasonable strategy in saving health resources.

While in diagnosing PCOS, BMI has better value as it is more specific. So the cut-off point of BMI at 26.6 kg/m^2^ can be used to facilitate the diagnosis of PCOS as it has a specificity of 98 %. It can be used as a relatively reliable diagnosis reference for PCOS when limited by equipment or economic situation, i.e., no ultrasound or sex hormones test available.

One disadvantage of this study is that the study subjects enrolled in this study were all from northern China, in the future, we still need to further expand the sample size, to summarize population data from multiple regions in China, and to establish a more accurate cut-off points in the prediction, screening and diagnosis of PCOS, as well as to administer weight loss treatment in obese PCOS patients to prevent long-term complications.

## Conclusion

To summarize, endocrine, reproductive and metabolic disorders are more severe in obese PCOS patients. BMI, WC and PBF are three important parameters to measure obesity. This study shows that patients with PCOS have serious overweight or obesity problems. WC, PBF and BMI are valuable in screening or diagnosing PCOS. PBF has a better sensitivity and can be used to screen PCOS, while BMI can be used in the diagnosis of PCOS as it has a better specificity.

## Abbreviations

ROC: the receiver operating characteristic
BMI: body mass index
WC: waist circumference
PBF: percentage of body fat
PCOS: polycystic ovary syndrome
LH: luteinizing hormone
ASRM: the American Society for Reproductive Medicine
SHBG: sex hormone-binding globulin
WHO: World Health Organization
IDF: the International Diabetes Federation
